# Hydrophobia

**Published:** 1869-02

**Authors:** 


Hydrophobia.—A Popular Medical Error.—An instructive article, if not a whole volume, might be written on the vulgar errors of physicians. None is more absurd or unfounded than that which supposes hydrophobia to be peculiar to, or more frequent in the hot months. The muzzling of dogs at such times is a relic of mediæval superstition. It originates from some popular notion concerning the "dog days," which in turn derive their name from the "dog star" Sirus. In point of fact hydrophobia is more frequent in January than in August, and to put muzzles on dogs in the summer season when they are so thirsty, is a piece of useless and wanton barbarity. We are glad to learn that the town of West Chester, in this State, propose to take the initiative in this matter, and do away with this antiquated habit. Let other and all towns follow this example.

